# A Novel Probiotic Mixture Exerts a Therapeutic Effect on Experimental Autoimmune Encephalomyelitis Mediated by IL-10 Producing Regulatory T Cells

**DOI:** 10.1371/journal.pone.0009009

**Published:** 2010-02-02

**Authors:** Shahram Lavasani, Balik Dzhambazov, Mehrnaz Nouri, Frida Fåk, Sophia Buske, Göran Molin, Henrik Thorlacius, Jan Alenfall, Bengt Jeppsson, Björn Weström

**Affiliations:** 1 Department of Cell and Organism Biology, Lund University, Lund, Sweden; 2 Department of Experimental Medical Science, Lund University, Lund, Sweden; 3 Surgery Research Unit, Clinical Research Centre, Department of Clinical Sciences, Lund University, Malmö, Sweden; 4 Department of Food Technology, Engineering and Nutrition, Chemical Center, Lund University, Lund, Sweden; 5 Probi AB, Lund, Sweden; New York University, United States of America

## Abstract

**Background:**

Multiple sclerosis (MS) is a chronic inflammatory autoimmune disease of the central nervous system (CNS). One potential therapeutic strategy for MS is to induce regulatory cells that mediate immunological tolerance. Probiotics, including lactobacilli, are known to induce immunomodulatory activity with promising effects in inflammatory diseases. We tested the potential of various strains of lactobacilli for suppression of experimental autoimmune encephalomyelitis (EAE), an animal model of MS.

**Methodology/Principal Findings:**

The preventive effects of five daily-administered strains of lactobacilli were investigated in mice developing EAE. After a primary screening, three *Lactobacillus* strains, *L. paracasei* DSM 13434, *L. plantarum* DSM 15312 and DSM 15313 that reduced inflammation in CNS and autoreactive T cell responses were chosen. *L. paracasei* and *L. plantarum* DSM 15312 induced CD4^+^CD25^+^Foxp3^+^ regulatory T cells (Tregs) in mesenteric lymph nodes (MLNs) and enhanced production of serum TGF-β1, while *L. plantarum* DSM 15313 increased serum IL-27 levels. Further screening of the chosen strains showed that each monostrain probiotic failed to be therapeutic in diseased mice, while a mixture of the three lactobacilli strains suppressed the progression and reversed the clinical and histological signs of EAE. The suppressive activity correlated with attenuation of pro-inflammatory Th1 and Th17 cytokines followed by IL-10 induction in MLNs, spleen and blood. Additional adoptive transfer studies demonstrated that IL-10 producing CD4^+^CD25^+^ Tregs are involved in the suppressive effect induced by the lactobacilli mixture.

**Conclusions/Significance:**

Our data provide evidence showing that the therapeutic effect of the chosen mixture of probiotic lactobacilli was associated with induction of transferable tolerogenic Tregs in MLNs, but also in the periphery and the CNS, mediated through an IL-10-dependent mechanism. Our findings indicate a therapeutic potential of oral administration of a combination of probiotics and provide a more complete understanding of the host-commensal interactions that contribute to beneficial effects in autoimmune diseases.

## Introduction

Multiple sclerosis (MS) is believed to be a T cell-mediated inflammatory autoimmune disease directed against myelin or oligodendrocytes in the central nervous system (CNS) and considered as one of the most common neurological diseases of young adults in Europe and North America [1]. Much progress has been made over the past decade in elucidating the causes of MS and directing therapies toward improving patient outcomes, but still there are no optimal therapies available aiming at slowing the disease progression [2]. Experimental autoimmune encephalomyelitis (EAE) in mice is an established animal model for MS sharing a number of clinical, genetic and immunological features with the human disease, which makes it suitable to elucidate the pathogenesis and devise therapy [3]. Failure or breakdown of immunological tolerance is believed to result in autoimmune disorders and CD4^+^CD25^+^ regulatory T cells (Tregs) have shown to be pivotal players in the maintenance of immune tolerance [4]. Several studies have indicated their role in the prevention of autoimmunity in animal models and evidence for disturbed or dysfunction of Tregs have also been observed in patients with different autoimmune diseases, including MS [5], [6]. Tregs are developmentally classified into natural, induced or adaptive populations. Most natural Tregs constitutively express the interleukin (IL)-2 receptor α chain (CD25), are selected in the thymus, and their development and function depend on the expression of the transcription factor forkhead box P3 (FOXP3) [5]. The mechanism of action of Tregs is not completely understood, but involves cell-cell contact and secretion of the immunoregulatory cytokines IL-10 and transforming growth factor (TGF)-β [7], [8]. Recent reports have shown that adoptive transfer of CD4^+^CD25^+^ Tregs significantly protected against EAE and decreased CNS inflammation through a mechanism that involves IL-10 [9], [10]. A key function of TGF-β and IL-10 is to maintain T cell tolerance to self by regulating differentiation and homeostasis of effector and regulatory T cells [11], [12]. Interestingly, it has also been shown that IL-10 expression by T cells is regulated by IL-27 and TGF-β [13].

Recent studies suggest that an impaired intestinal barrier function might cause an imbalance between Th1 and Th2 immune responses, which in turn, can trigger autoimmune processes [14]. *Lactobacillus* species with probiotic properties are often also part of the commensal microflora of the intestinal tract in humans and animals and are generally recognized to confer beneficial health effects [15]. The immunomodulatory properties of probiotics have raised a lot of interest in recent years. These include regulation of intestinal microbial homeostasis, maintenance of the gastrointestinal barrier function, interference with the ability of pathogens to colonize [16], [17], [18], [19], [20], [21], [22] and finally modulation of local and systemic immune responses [23]. The mechanisms underlying these beneficial effects are not completely understood but have been associated with immunomodulatory properties of specific probiotic strains [24]. Probiotics can reverse immunological disturbances by inducing immunoregulatory responses mediating a control of the balance between pro- and anti-inflammatory cytokines [25], [26]. Reviews of several clinical studies support that probiotics may represent a capable preventive and therapeutic strategy for allergic and chronic inflammatory diseases [24], [27]. The anti-inflammatory effect of probiotics has been attributed to increased production of IL-10 by immune cells in the lamina propria, Peyer's patches and the spleen of treated animals [26], [28]. Moreover, recent studies provided evidence that one effect of probiotics may involve induction of differentiation of IL-10-dependent, TGF-β-bearing regulatory cells [26], [29]. Therefore, probiotics have emerged as promising candidates for treatment of inflammatory and autoimmune disorders.

In the present study, we investigated the preventive effects of five daily-administered potential probiotic strains (two strains of *Lactobacillus (L) paracasei*, two strains of *L. plantarum* and the traditional yoghurt bacterium *L. delbrueckii*, subsp. *bulgaricus*) comparatively on EAE development in mice. After a primary screening, three strains, *L. paracasei* DSM 13434, *L. plantarum* DSM 15312 and *L. plantarum* DSM 15313, that efficiently prevented EAE development were chosen for further evaluation. We found that the immunosuppressive potential of these probiotic strains was associated with induction of Tregs and production of IL-4, IL-10 and TGF-β1 in mesenteric lymph nodes (MLNs) and spleen. Despite a preventive effect on EAE, administration of each individual strain to mice with established EAE was not capable to suppress the disease. Previous research works with probiotic strains have suggested synergistic effects of combinations of probiotic lactobacilli with specific properties [30], [31]. We therefore investigated this using different combination of our chosen lactobacilli which resulted in a mixture containing all three probiotic lactobacilli (Lacto-mix). We showed that therapeutic treatment with this new probiotic mixture successfully reversed established EAE and demonstrated a unique synergistic effect of these strains to regulate systemic IL-10 release and induce functional Tregs in, not only intestinal lymph nodes, but also in the periphery and CNS of diseased animals. Our results emphasize the usefulness of the EAE model to select potential probiotic strains to design a mixture of disease specific probiotics and finally the potential of a novel probiotic lactobacilli mixture for therapeutic intervention in inflammatory diseases of the CNS.

## Results

### Oral Administration of Some Strains of *Lactobacillus* Prevents EAE

**Figure 1:**
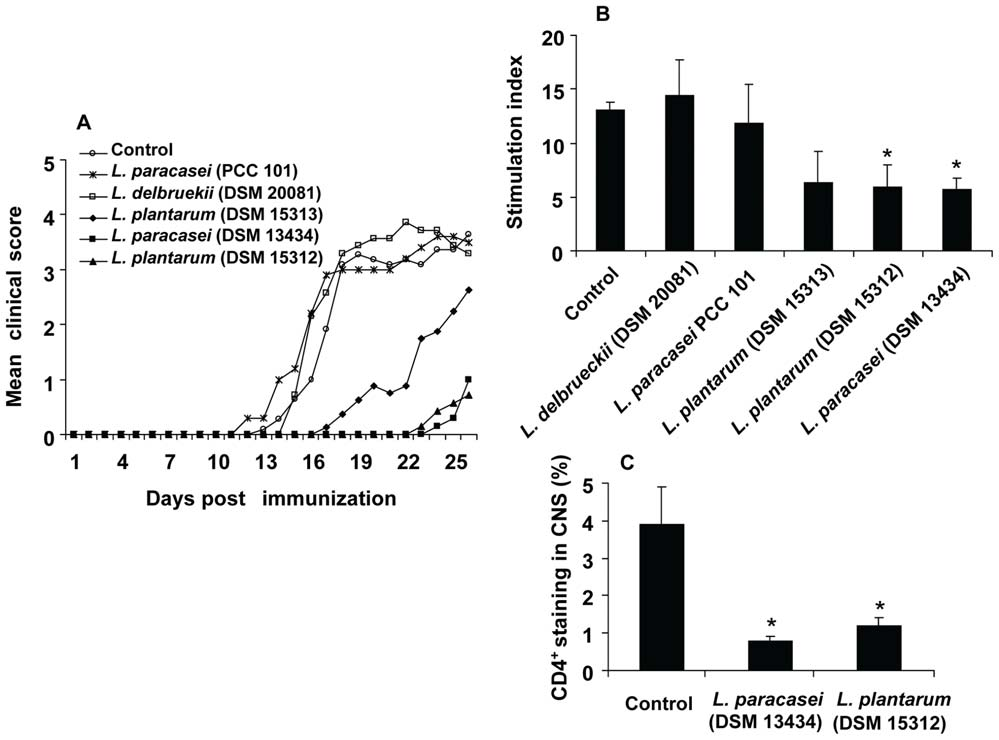
Distinct probiotic strains prevents EAE and suppresses MOG-reactive T cells. C57BL/6 mice received either *L. paracasei* DSM 13434, *L. plantarum* DSM 15312, *L. plantarum* DSM 15313 *L. paracasei* PCC 101, *L. delbrueckii* DSM 20081 (10^9^ cfu, daily) or vehicle as control, starting 12 days prior to immunization for EAE. (A) The mean clinical score for each group of mice (n = 8) is shown. Data are representative of three separate experiments. (B) Spleen cell cultures from the probiotic-treated or control mice were restimulated *in vitro* with MOG_35–55_ peptide. MOG specific T cell proliferation was conducted with [^3^H]thymidine incorporation assay and each bar represents the mean stimulation index (± SEM) from triplicate measurements (n = 3). (C) Immunohistochemical staining for CD4^+^ T cells in sections from spinal cord isolated from *L. paracasei* DSM 13434-, *L. plantarum* DSM 15312-treated and control EAE animals at day 25 of immunization. In the histogram, each bar represents the mean percentage of stained area (± SEM). * represents a p-value≤0,05.

Targeting the immune response in the periphery has led to a number of therapies for MS, however the serious side effects of the drugs have prevented their extensive clinical applications [2]. Although the immunomodulatory potential for some probiotic strains has been investigated mostly in inflammatory bowel diseases and allergic disorders, little is known regarding the desired probiotic properties for extraintestinal autoimmune diseases, in particular for the treatment of MS. Moreover, many probiotic preparations have been tested in several laboratories with diverse and sometimes contradictory results [24]. In order to examine the ability of probiotic lactobacilli to affect systemic immune responses by suppressing the T cell-mediated chronic inflammation in the CNS, five different strains, *L. paracasei* DSM 13434, *L. plantarum* DSM 15312, *L. plantarum* DSM 15313 *L. paracasei* PCC 101, *L. delbrueckii* DSM 20081 or vehicle as control, were orally administered daily to groups of C57BL/6 mice, starting 12 days prior to immunization for EAE. We observed that treatment with *L. paracasei* DSM 13434, *L. plantarum* DSM 15312 or *L. plantarum* DSM 15313 prevented and delayed the onset of the clinical signs of EAE compared to control mice, although efficient prevention was most extensive in mice receiving *L. paracasei* DSM 13434 or *L. plantarum* DSM 15312 (Fig. 1A). In contrast, treatment with *L. paracasei* PCC 101 and *L. delbrueckii* DSM 20081 had no effect on the disease development (Fig. 1A).

Due to the fact that activation of autoreactive CD4^+^ T cells followed by specific recruitment into the CNS appears fundamental for EAE development [32], we further analysed the proliferative responses and CNS infiltration of disease related T cells in animals treated with probiotics. *Ex vivo* analysis of splenocytes, obtained 25 days after the immunization, showed significantly decreased MOG-reactive T cell proliferation in animals fed with *L. paracasei* DSM 13434, *L. plantarum* DSM 15312 or *L. plantarum* DSM 15313 compared to splenocytes from animals fed with *L. paracasei* PCC 101, *L. delbrueckii* DSM 20081 or control mice (Fig. 1B). These data clearly demonstrated the unique capacity of the probiotic strains, in particular *L. paracasei* DSM 13434 and *L. plantarum* DSM 15312 to ameliorate development of chronic EAE and a direct correlation in their ability to suppress the disease-inducing inflammatory T cells. Drastically reduced infiltration of CD4^+^ T cells in CNS tissues of these mice, measured by immunohistochemical analysis, further demonstrated the protective effect of these probiotic strains (Fig. 1C).

### *L. paracasei* Treatment Reduces Pro-Inflammatory Cytokine Expression

**Figure 2:**
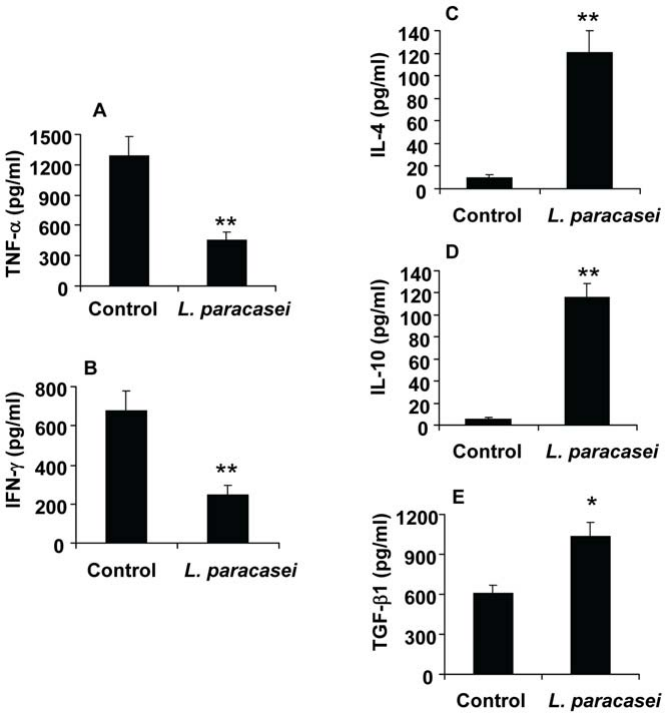
*L. paracasei* reduces secretion of pro-inflammatory and promotes secretion of anti-inflammatory cytokines in EAE mice. (A–E) Spleen cell cultures from probiotic-treated or control mice were restimulated *in vitro* with MOG_35–55_ peptide, supernatants were collected and the levels of pro-inflammatory Th1 cytokines, TNF-α and IFN-γ, and Th2 cytokines, IL-4, IL-10 and TGF-β1, were determined using ELISA kits. Data are representative of two experiments. Each bar represents mean ± SEM of three samples per group. * represents a p-value ≤0,05 and ** a p-value ≤0,01.

The ability of probiotic bacteria to relieve inflammation has in many studies been related to increased elaboration of regulatory cytokines in the gastrointestinal tract [23]. In addition, little is known about their effect on systemic cytokine responses. In order to investigate the mechanism behind the systemic immunosuppressive effect of our probiotic strains, splenocytes isolated from EAE animals treated with *L. paracasei* DSM 13434, were examined for expression of a panel of different cytokines. *In vitro* analysis of supernatants collected from MOG-stimulated splenocyte cultures showed significantly decreased secretion of the pro-inflammatory cytokines TNF-α and IFN-γ from cells of probiotic-treated mice, at day 25 post immunization, compared to control mice (Fig. 2A and B). In contrast, the release of Th2 type cytokines, IL-4, IL-10 and TGF-β1, was highly enhanced in spleen cell cultures from *L. paracasei* treated animals (Fig. 2C–E). Similar results were also achieved from splenocytes isolated from *L. plantarum* DSM 15312 treated mice (unpublished data). Taken together, these data suggest that the suppressive effect of probiotic treatment on disease-related peripheral Th1 cells is associated with a shift of cytokine profile from a Th1 type toward a Th2 type response (including anti-inflammatory cytokines IL-10 and TGF-β1).

### Treatment with a Combination of Three Selected Probiotic Lactobacilli Reverses Established EAE

**Figure 3:**
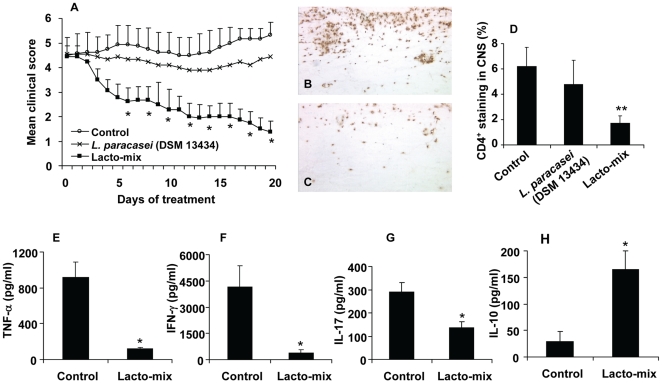
Administration of three potential probiotic strains suppresses inflammatory autoimmune disorders in mice with established EAE. C57BL/6 mice were immunized and scored for clinical signs of EAE. The animals were orally treated, starting 14 days after the onset of EAE, with *L. paracasei* DSM 13434 (n = 18), a mixture of lactobacilli (Lacto-mix) containing *L. paracasei* DSM 13434, *L. plantarum* DSM 15312 and DSM 15313 (n = 18) or saline (vehicle) as control (n = 18). (A) The mean clinical score for each group of mice is shown. Data represent pooled values from two independent experiments. The presence of infiltrating CD4^+^ T cells into the spinal cord parenchyma and perivascular cuffs was assessed by immunohistochemical staining in sections from (B) control EAE and (C) Lacto-mix-treated animals after 20 days of therapeutic treatment. (D) In the histogram, each bar represents the mean percentage of stained area (± SEM) from 5 mice per treatment group. Levels of pro-inflammatory cytokines (E) TNF-α, (F) IFN-γ, (G) IL-17, and (H) anti-inflammatory IL-10 were measured in supernatants of MOG_35–55_ peptide restimulated spleen cell cultures from the Lacto-mix-treated (n = 3) or control mice (n = 3), after 20 days of therapeutic treatment. Data are representative of two experiments. Each bar represents mean ± SEM of three samples per group. * represents a p-value ≤0,05 and ** a p-value ≤0,01.

We have demonstrated that oral administration of *L. paracasei* DSM 13434, *L. plantarum* DSM 15312, *L. plantarum* DSM 15313 prevented and delayed the onset of EAE (Fig. 1). We have also shown that *L. paracasei* DSM 13434 and *L. plantarum* DSM 15312 induced release of the Th2 type cytokines IL-4, IL-10 and TGF-β1 in spleen cells from EAE mice (Fig. 2). The major goal of our approach was to find an appropriate immunosuppressive therapy in the treatment of MS in patients with already developed clinical signs or symptoms. Therefore, we tested the therapeutic efficacy of our probiotic treatments in established EAE. Mice were individually fed with *L. paracasei* DSM 13434, *L. plantarum* DSM 15312, or *L. plantarum* DSM 15313 every second day, starting 14 days after onset of EAE while a control group received saline. We observed that oral administration of a monostrain probiotic had no major effect on disease progression. A representative treatment with *L. paracasei* DSM 13434 is shown in Figure 3A. Probiotic strains exist with a broad spectrum of health improving effects and a combination of strains with specific properties has been suggested to induce wider antimicrobial spectrum and stronger anti-inflammatory effects [30], [31]. Thus, we hypothesized that synergistic beneficial effects may be achieved by treatment with combinations of the three chosen probiotic strains with protective properties against EAE. Oral administration with mixture of two strains (randomly mixed) had no measurable suppressive effect on established chronic EAE (unpublished data). However, a mixture of all three strains of *L. paracasei* DSM 13434, *L. plantarum* DSM 15312 and *L. plantarum* DSM 15313 successfully suppressed and reversed chronic EAE in contrast to the saline fed (Fig. 3A). This mixture is subsequently referred to as “Lacto-mix” herein. Further, clinical improvement by treatment with the Lacto-mix was directly associated with reduced CNS inflammation and infiltration of disease-related CD4^+^ T cells (Fig. 3B–D). In addition, analysis of the cytokine profile released by spleen cells isolated from Lacto-mix-treated animals also showed significantly decreased secretion of inflammatory TNF-α, IFN-γ and IL-17 by MOG-reactive T cells compared to cells from control EAE mice (Fig. 3E–G). Conversely, production of anti-inflammatory IL-10 was markedly enhanced by MOG-reactive spleen T cells in mice treated with this probiotic combination (Fig. 3H). IL-4 and TGF-β1 production by spleen cells in Lacto-mix-treated mice was not statistically different from that observed in control EAE mice (unpublished data).

In an attempt to examine the differential adjuvanticity response to live or killed lactobacilli strains, heat killed Lacto-mix was also used for treatment of EAE mice. However, the therapeutic effect was not reproduced and no striking differences on clinical signs of EAE were observed in treated versus control diseased animals (unpublished data).

These data indicate that a combination of three live strains of *Lactobacillus* (Lacto-mix), with evident inhibitory properties against EAE, is inducing a synergistic effect regarding the capacity to induce IL-10, suppression of the inflammatory cytokines and the therapeutic potential in diseased animals.

### Probiotic Treatment Induces Regulatory T Cells in an IL-10-Dependent Manner

**Figure 4:**
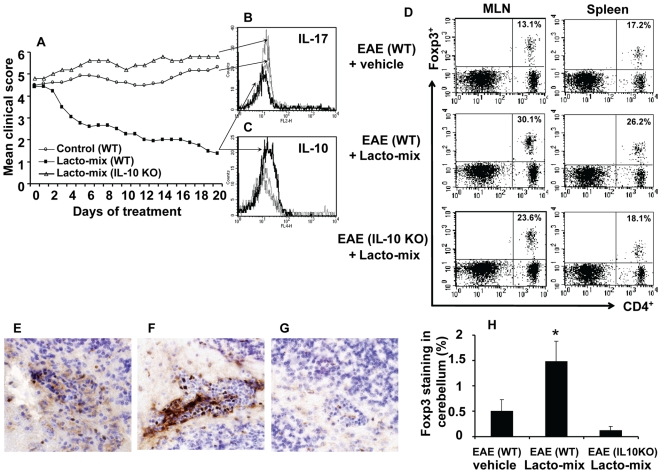
Probiotic treatment suppresses IL-17 expression and favors Treg emergence in an IL-10-dependent manner. C57BL/6 wild type (WT) or IL-10-KO mice were immunized and observed for clinical signs of EAE. Fourteen days after the onset of EAE, animals were orally treated with a mixture of lactobacilli (Lacto-mix) or saline as control. (A) The mean clinical score for each group of mice is shown. Data represent pooled values from two independent experiments. Histograms show the expression of (B) IL-17 and (C) IL-10 by CD4^+^ T cells freshly isolated from the brain of Lacto-mix-treated WT (thick line), IL-10 KO (dashed line), or saline-treated control mice (thin line), after 20 days of therapeutic treatment. (D) MLNs and spleens isolated from these animals were analysed by flow cytometry for expression of CD4, CD25 and Foxp3. Presented FACS plots show the percentage of CD4^+^Foxp3^+^ cells of the total CD4^+^ T cells (numbers in quadrants) in control EAE (top panel), Lacto-mix-treated WT (middle panel), or IL-10 KO (bottom panel) mice. The presence of infiltrating Foxp3^+^ cells into the cerebellum parenchyma and perivascular cuffs was assessed by immunohistochemical staining of sections from (E) control EAE, (F) Lacto-mix-treated WT, (G) Lacto-mix-treated IL-10 KO. (H) In the histogram, each bar represents the mean percentage of Foxp3 stained area (± SEM) from 3 mice per treatment group. Data are representative of two experiments. * represents a p-value ≤0,05.

Our findings suggested that a treatment with the Lacto-mix was therapeutic and induced production of IL-10 in activated T cells (Fig. 3). There is also accumulated information suggesting that certain probiotics can act through induction of regulatory T cells that suppress inflammation-provoking effector cells [23]. In order to better understand how our chosen lactobacilli strains regulate the immunosuppressive responses and to investigate whether this was mediated by IL-10 or regulatory T cells, we further investigated EAE mice treated with the Lacto-mix including IL-10-defcient mice. We demonstrated that the therapeutic activity of probiotics was completely abolished in EAE mice lacking IL-10 (Fig. 4A). Analysis on CD4^+^T cells isolated from CNS revealed drastically decreased amounts of IL-17 producing CD4^+^T cells in wild-type (WT) EAE mice receiving the Lacto-mix which instead showed a significant upregulation of IL-10 producing CD4^+^T cells (Fig. 4B and C). Since our probiotic treatment inhibited all inflammatory responses during EAE following the activation of IL-10, there is an indication that this therapeutic action is inducing Tregs with suppressor activity during the progression of the disease. Therefore, we investigated cells isolated from MLNs and spleen of the Lacto-mix-treated mice and when compared to control EAE animals, we found a significant increase in percentage and absolute numbers of CD4^+^ cells co-expressing CD25 and Foxp3 in MLNs of both probiotic treated WT and IL-10 KO, but only in spleens of WT animals (Fig. 4D).

The accumulation of IL-10 producing Treg cells in CNS of EAE mice has been shown to be correlated to natural recovery from the disease [33]. We examined brain tissues isolated from Lacto-mix-treated WT animals and found significant increased numbers of Foxp3^+^ cells in CNS (mainly localized in the perivascular cuffs of cerebellum), concomitant to the recovery of EAE (Fig. 4, E–H). The changes in Foxp3^+^ cells in probiotic treated WT animals were uniquely followed by an increase of CD4^+^Foxp3^+^ cells in the spleen of these mice which also correlated with increased amounts of IL-10 producing CD4^+^ cells in the CNS. These data strongly suggest that our chosen mixture of three lactobacilli strains induces the generation of activated Treg cells in the intestinal lymphoid organs and that the emergence of probiotic induced Tregs in spleen and CNS of diseased animals is dependent on IL-10.

### Probiotic Specific Induction of Cytokines and Tregs

**Figure 5:**
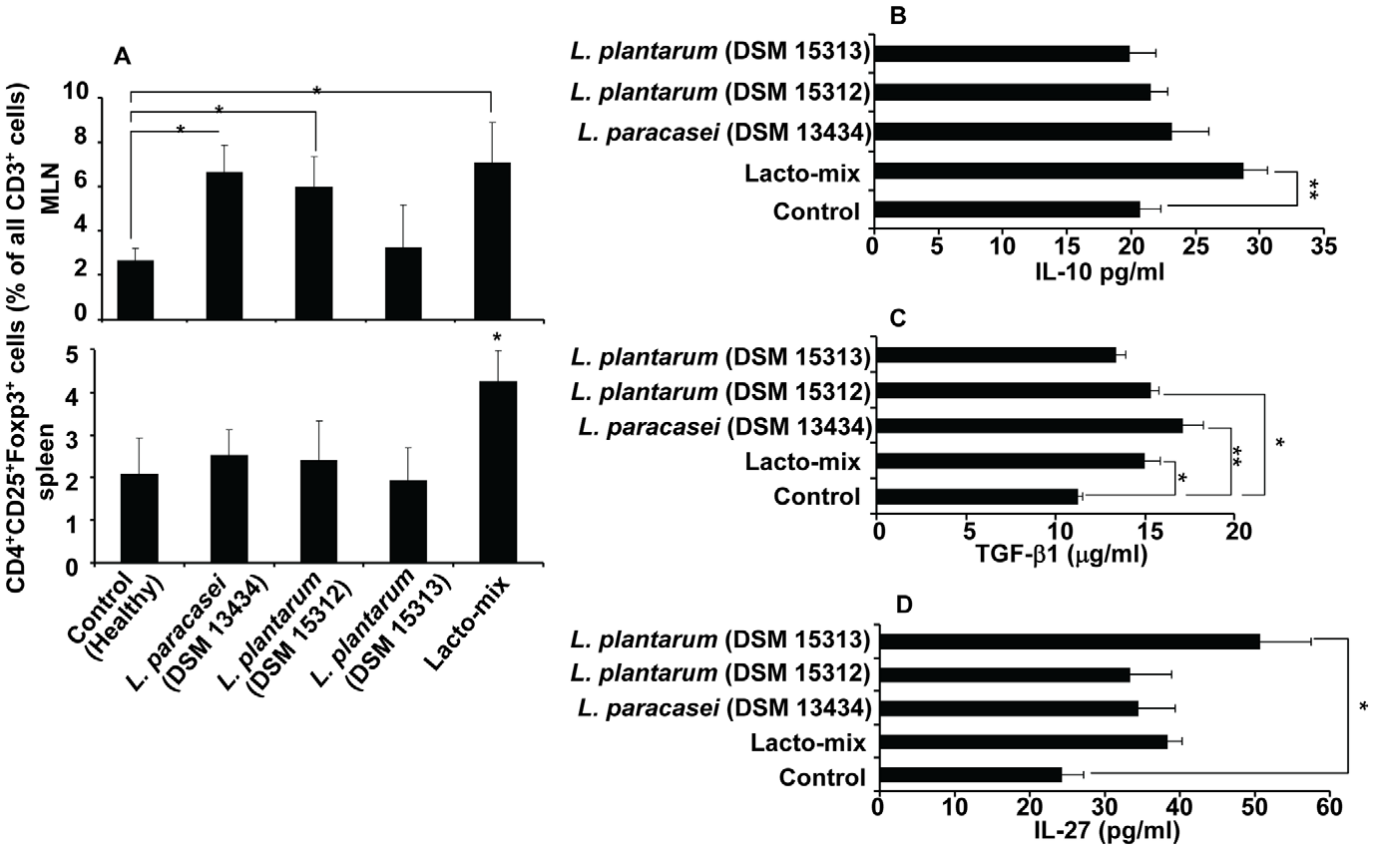
A synergistic combination of probiotics induces Treg cell expansion and systemic IL-10 production. Healthy C57BL/6 mice were daily fed with *L. paracasei* DSM 13434, *L. plantarum* DSM 15312, *L. plantarum* DSM 15313, a mixture of all three (Lacto-mix) or saline (control) during 14 days (n = 5). MLNs and spleens isolated from these animals were analysed for expression of CD3, CD4, CD25 and Foxp3 by flow cytometry. (A) The proportion of CD4^+^CD25^+^Foxp3^+^ stained cells was examined on the total gated CD3^+^ lymphocytes from MLNs (top) and spleen cells (bottom). Blood serum from these animals was analysed for different cytokines using ELISA kits. Shown are data from (B) IL-10, (C) TGF-β1 and (D) IL-27 analysis. Data are mean ± SEM and representative of two experiments. * represents a p-value ≤0,05 and ** a p-value ≤0,01.

We showed that treatment with *L. paracasei* DSM 13434, *L. plantarum* DSM 15312 or *L. plantarum* DSM 15313 can prevent EAE development in mice, but only a combination of all three lactobacilli resulted in an efficient therapeutic activity when given to mice with established disease (Fig. 3A). Our results demonstrated the ability of these strains to modulate both local and systemic immune responses with a strong potential to suppress autoreactive effector T cells in the periphery and highlighted the induction of Tregs and immunoregulatory cytokines IL-10 (Fig. 4). There is emerging evidence that oral administrations of certain probiotics are able to induce Tregs locally in the gastrointestinal tract [23]. It is also known that the function of Tregs depends on IL-10 and/or TGF-β [34]. These findings raises a question whether the regulatory T cells, once differentiated in the gut, are either migrating out to the periphery or the systemic cytokine release, induced by probiotics, can favor differentiation of regulatory cell population outside the gut. To investigate whether there are proportional differences in Treg subsets induced by different lactobacilli, groups of healthy C57BL/6 mice were fed with *L. paracasei* DSM 13434, *L. plantarum* DSM 15312, *L. plantarum* DSM 15313, a combination of all three (Lacto-mix), or vehicle (control) for 14 days. MLNs and spleen were collected and analysed for CD4^+^ T cell subsets expressing the Treg markers CD25^+^ and Foxp3^+^ protein. From analysis of MLNs, we found significantly increased proportions of Tregs in animals treated with *L. paracasei* DSM 13434, *L. plantarum* DSM 15312 or the Lacto-mix. Interestingly, analysis of the spleens collected from these animals showed significant higher frequency of Tregs only in mice receiving the Lacto-mix (Fig. 5A).

In order to determine whether cytokines released in the peripheral blood of these probiotic treated mice correlated with the systemic immunomodulatory effect of the strains, we further investigated the presence of different cytokines in blood collected from these mice. Serum samples were analysed for a panel of cytokines, including IL-10, TGF-β1 and IL-27 by ELISA. A significant increase of IL-10 was only observed in serum samples from animals treated with the Lacto-mix but not when exposed to the probiotic monostrains, compared to control mice (Fig. 5B). On the other hand, cytokines analysed in serum from mice treated with monostrain probiotic showed significantly increased levels of TGF-β1 in *L. paracasei* DSM 13434 and *L. plantarum* DSM 15312 treated animals while elevated IL-27 serum levels were only measured in mice fed with *L. plantarum* DSM 15313 (Fig. 5C and D). Serum levels of TNF-α, IFN-γ, IL-17 or IL-4 were found to be undetectable or of no significant difference in probiotic treated versus control animals (unpublished data). Interestingly, recent studies have shown that IL-27 limits Th1 and Th2 responses and enhances IL-10 production by T cells, which can be further enhanced in presence of TGF-β [35]. These findings suggest a synergistic effect of the three chosen probiotic strains regarding their capacity to induce serum IL-10 which seems to be of importance for emergence of probiotic induced Tregs in the periphery.

### The Immunosuppressive Effect of Probiotic Induced Tregs Is Transferable and IL-10-Dependent

**Figure 6:**
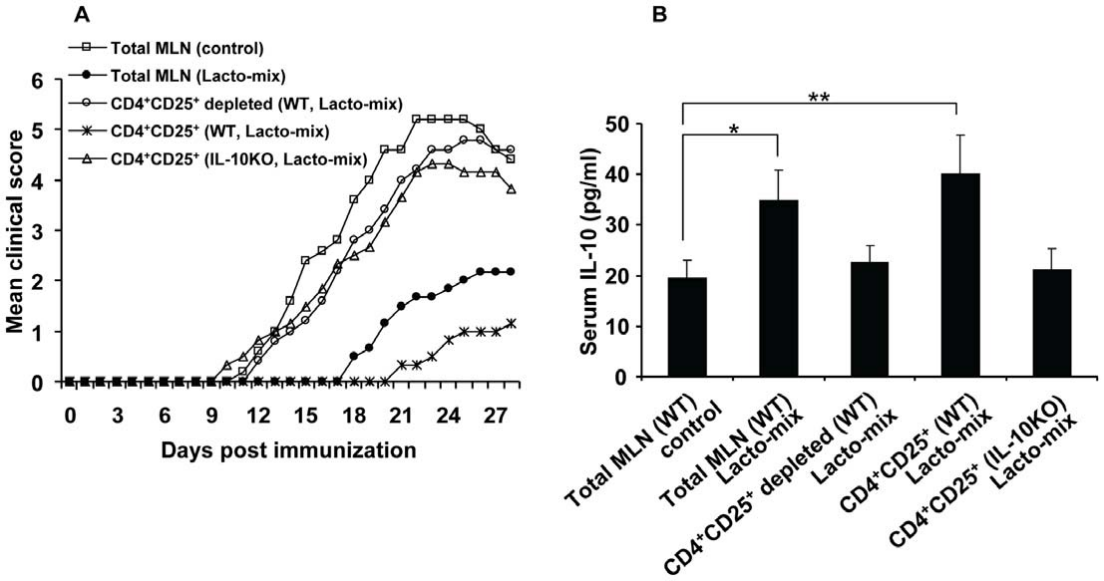
Probiotic-induced Treg cells suppress EAE in an IL-10-dependent fashion. Naïve C57BL/6 wild type (WT) or IL-10-KO mice were treated with either the probiotic Lacto-mix or saline (control) during 14 days. MLN cells were isolated from these animals and administered intravenously into recipient mice which were immunized for EAE 24 h later. (A) EAE development in recipient mice after adoptive cell transfer of total MLN cells (isolated from mice treated with either saline or Lacto-mix), MLNs (isolated from WT mice treated with Lacto-mix) depleted for CD4^+^CD25^+^ cells, isolated CD4^+^CD25^+^ cells (from MLNs of Lacto-mix treated WT mice), isolated CD4^+^CD25^+^ cells (from MLNs of Lacto-mix treated IL-10 KO mice) (n = 3−5). (B) Serum levels of IL-10 were measured by ELISA in plasma obtained from recipient animals at 28 days after cell transfer. Data are representative of two experiments. Each bar represents mean ± SEM of three samples per group. * represents a p-value ≤0,05 and ** a p-value ≤0,01.

Our data demonstrated that administration of the Lacto-mix induces IL-10 and support the induction of Treg populations in lymphoid organs (Fig. 5). To assess the tolerogenic activity of probiotic induced Treg cells and the significance of their IL-10 expression, we tested whether this effect is transferable to naïve mice. WT and IL-10 KO mice were orally fed with Lacto-mix or vehicle (control) daily, during 14 days. MLN cells from treated animals were isolated and adoptively transferred into naïve WT mice which were induced for EAE one day later. The animals were then followed for clinical signs of the disease. The result showed that the EAE symptoms were markedly suppressed in animals which received MLN cells from WT mice fed with Lacto-mix compared to those which received MLN cells from control WT mice (Fig. 6A). To investigate the involvement of Tregs, MLN cells isolated from Lacto-mix-treated WT mice were subjected to selective depletion of CD4^+^CD25^+^ T cell populations before transfer to naïve recipients which then were immunized for EAE. Evaluation of these animals clearly revealed that depletion of CD4^+^CD25^+^ T cells from the MLN cell population abolished the suppressive effect on EAE development (Fig. 6A). To further assess the regulatory activity of probiotic induced Tregs, purified CD4^+^CD25^+^ T cell from MLNs of WT or IL-10 KO mice were adoptively transferred into naïve WT mice. As shown in Figure 6A, EAE development in mice receiving CD4^+^CD25^+^ T cells from WT probiotic-treated animals was strongly delayed and suppressed compared to those receiving CD4^+^CD25^+^ T cells from treated IL-10 KO or WT control mice. Additional analysis of these animals showed that serum levels of IL-10 in mice receiving isolated MLNs or CD4^+^CD25^+^ T cells from probiotic-treated mice were significantly increased compared to mice receiving MLN cells from treated IL-10 KO mice or control donors and this effect was completely abrogated in mice receiving CD4^+^CD25^+^ T cells depleted MLN cells of probiotic fed WT mice (Fig. 6B). Taken together, these data provide evidence that IL-10 producing CD4^+^CD25^+^ T cells are involved in the protective effect induced by treatment with the combination of our three chosen probiotic strains.

## Discussion

Autoimmune diseases, such as MS, may result from the failure of tolerance mechanisms to prevent expansion of inflammatory autoreactive T cells. CD4^+^ Treg cells are considered as key mediators in the maintenance of immune tolerance and potent modulators of T cell-mediated immune responses [5]. Treg populations have been classified into natural CD4^+^CD25^+^Foxp3^+^ Tregs and induced or adaptive Tregs [36]. Many studies suggest the importance of regulatory cytokines IL-10 and TGF-β in mediating the suppressive activity of Tregs [13]. Accordingly, the functional impairment and diminished Foxp3 expression of Treg cells in MS patients [37], [38] seem to be directly correlated with decreased expression of immunosuppressor cytokine IL-10 released by T cells [39]. Moreover, the ability of Treg cells to mediate spontaneous recovery from an active disease process in EAE and MS patients suggest the therapeutic potential of a possible restoration of Treg homeostasis [6], [33].

Recent interests in the influence of intestinal microflora and gut barrier function on chronic inflammatory and autoimmune disease have resulted in efforts to improve the microfloral composition by using probiotics [14], [26], [40], [41]. There is emerging evidence that certain probiotic bacterial strains exert anti-inflammatory effects by the ability to favor induction of IL-10-dependent, TGF-β-bearing regulatory cells [23], [26], [28]. Although strain-dependent effects of probiotics in controlling the inflammatory diseases have been suggested, the essential molecular mechanism of these bacteria has not been elucidated yet [42], [43]. Therefore, a better understanding of the protective role of different probiotics may provide ways to use them to induce regulatory cell responses sufficient to treat severe systemic autoimmunity.

In our study, we have identified three potential probiotic strains with novel immunoregulatory properties on chronic CNS inflammation and designed a unique multispecies combination of *Lactobacillus* with a therapeutic potential in established chronic EAE.

Initially, we tested five different *Lactobacillus* strains with known probiotic activity for their ability to protect against EAE. We found that oral administration of three of these strains, *L. paracasei* DSM 13434, *L. plantarum* DSM 15312 and *L. plantarum* DSM 15313, prior to immunization, delays the onset and suppresses the progression of EAE. Our data revealed that the suppressed activity was associated with reduced inflammation in CNS, down-regulation of MOG_35-55_-induced T cell responses and a shift in production of pro-inflammatory Th1 cytokines toward the beneficial Th2 type response including IL-4, IL-10 and TGF-β1. It is well known that EAE is mediated by Th-17 and Th1 cells secreting pro-inflammatory cytokines, such as IL-17, TNF-α and IFN-γ, while disease recovery is associated with increased levels of Th2 cytokines IL-4, IL-10 and TGF-β1 [44], [45]. These cytokines are known to suppress EAE by shifting the immune response from a Th1 to a Th2 response [46], [47], [48]. Thus, an upregulation of these cytokines observed in probiotic treated animals seems to induce a shift towards Th2 lymphocytes which may be one of the mechanisms of down-regulation of the autoimmune responses. In addition, IL-10 and TGF-β are potent regulatory cytokines acting directly on the differentiation and homeostasis of effector and Treg cells [13], [49]. In fact, despite of the Th2 cells, the major source of IL-10 and TGF-β are Treg populations [50], [51]. Therefore, we investigated the presence of CD4^+^CD25^+^Foxp3^+^ Tregs in naïve mice and found that administration of probiotics increased the numbers of these cells in MLN and spleen. More direct support was also achieved by showing significantly higher levels of IL-4, IL-10 and TGF-β1 in probiotic-treated mice.

Thus, it may be suggested that gut exposure to probiotic strains reduces autoreactive T cell activities by direct or indirect activation of regulatory CD4^+^ T cells. The mucosal environment of the gut is a unique environment for crosstalk between the commensal bacteria and the mucosal immune system which seems to affect immunological tolerance and homeostasis within the gut for priming of inflammatory T cells but also for activation of regulatory T cell populations [52]. Although the commensal flora is known to stimulate the mucosal immune cells, an increasing amount of evidence suggests that Treg cells induced by commensal bacteria are actively involved in peripheral tolerance, as well as in local tolerance but the real mechanism is to be elucidated [52]. Oral tolerance induced by anti-CD3 administration has been shown to suppress EAE by inducing TGF-β-dependent Tregs in the gut which inhibit the systemic autoimmune inflammation in EAE [53]. The concept that probiotics act via the induction of Treg populations has also been indicated in other inflammatory models. For example, a mixture of probiotic strains (VSL#3) ameliorates Th1-mediated murine colitis by inducing TGF-β-bearing Tregs and also suppresses diabetes development in NOD mice by inducing IL-10-producing cells in gut associated lymphoid tissues (GALT) [26], [28]. In fact, the immunosuppressive effect of probiotics used in these models is likely to occur when applied early in disease development. VSL#3 contains, in addition to bifidobacteria, a combination of several species of lactobacilli, in different proportions. However, little is yet known concerning the contributions of each strain to the VSL#3-mediated effects. Indeed, recent reports have emphasised both additive and synergistic probiotic effects but care has to be taken to explore each strain specifically to select potential candidates to design a multispecies and disease specific probiotic mixture [30], [31].

A major challenge in the search for an ideal immunotherapy for MS is that the clinical signs of disease are a consequence of an already established chronic inflammatory disorder of the CNS induced by activated immune cells. Considering this fact, a novel immunotherapy combining maximal efficacy and minimal side effects seeks to alter these inflammatory responses for treatment or prevention of the disease. In order to further analyse the immunosuppressive potential of our chosen probiotic strains on EAE, mice were immunized with MOG peptide and 14 days after the onset, when showing severe symptoms of the disease, they were orally fed with each chosen *Lactobacillus*. Notably, this approach was not as effective as our prophylactic treatment. To investigate possible synergistic effects of a combination of these strains in suppression of EAE, we repeated the experiment and fed the highly diseased animals with the mixtures of three chosen probiotic strains. Treatment with a random combination of only two different *Lactobacillus* strains did not induce a sufficient suppression of the disease. However, a mixture of the three probiotic strains, *L. paracasei* DSM 13434, *L. plantarum* DSM 15312, *L. plantarum* DSM 15313 (Lacto-mix), successfully inhibited the progression of EAE and significantly suppressed and reversed chronic EAE in diseased animals. Concurrently, splenocytes of the Lacto-mix-probiotic treated mice produced lower levels of inflammatory cytokines IL-17, TNF-α and IFN-γ, but increased amounts of IL-10, when restimulated *in vitro* with the autoantigen. In addition, clinical improvement of EAE mice was directly associated with suppressed inflammation and reduced amounts of disease-related CD4^+^ T cell infiltrates in CNS of Lacto-mix-treated mice.

As suggested by other studies it remains possible that probiotics have several means of exerting anti-inflammatory effects by improving epithelial cell function and induction of regulatory cells [23]. Nevertheless, these studies did not elucidate the crosstalk between the systemic immune responses and mucosal immune system in gut. Consequently, we analysed cytokine expression in the blood of probiotic-treated animals to determine any possible correlation with the disease process. Our results showed a unique strain-dependent pattern of cytokine formation, where *L. paracasei* DSM 13434 and *L. plantarum* DSM 15312 increased levels of TGF-β1 in the blood, while *L. plantarum* DSM 15313 enhanced blood levels of IL-27. Surprisingly, significantly increased serum levels of IL-10 were only measured in mice receiving a mixture of all three probiotic strains. Accordingly, the preventive effect of *L. paracasei* DSM 13434 and *L. plantarum* DSM 15312 against EAE development can further be explained. The important impact of TGF-β signalling in restoring the autoreactive T cell responses in peripheral tissues has already been shown in many studies [11]. Recent observations also suggest that TGF-β induces Foxp3 expression and differentiation of adoptive populations of Treg cells in peripheral tissues and GALT, in particular, and in excessive levels TGF-β can expand Tregs to protect against autoimmunity as it was demonstrated in diabetic NOD mice [13], [54]. Furthermore, these studies showed that retinoid acid released by GALT CD103^+^ dendritic cells further promote TGF-β-induced Treg cell differentiation which provide an important mechanism in T cell tolerance against commensal bacteria.

On the contrary, *L. plantarum* DSM 15313 showed to be a potent inducer of IL-27 in the present study. IL-27 is produced mostly by innate immune cells and to induce suppressive effects on Th1, Th2, and Th17 cell responses in various infection and autoimmune disease models [55]. IL-27 has also been shown to trigger IL-10 production by T cells and this can further be enhanced in presence of TGF-β [35]. This interesting finding may explain the observation of increased IL-10 blood levels in animals treated with the mixture of probiotics. Nevertheless, *L. paracasei* DSM 13434 and *L. plantarum* DSM 15312 are able to induce IL-10 in peripheral tissues but this may not be sufficient to increase cytokine levels in blood. In addition, recent studies on probiotics have revealed that they inhibit the generation of Th1 cells by promoting the appearance of both tolerance-inducing, IL-10-producing dendritic cells (DCs) and Treg cells [29]. IL-10 has been considered as a main anti-inflammatory cytokine with various effects on different cell populations [12]. Antigen stimulation in presence of this cytokine has been shown to favor IL-10 and TGF-β producing cells with immunoregulatory potentials on inflammatory disorders like EAE [56]. Taken together, we suggest that the additive and synergistic effect of our probiotic mixture can reveal an interrelation between TGF-β induced by *L. paracasei* DSM 13434 and *L. plantarum* DSM 15312 and IL-27 triggered by *L. plantarum* DSM 15313 to enhance promotion of IL-10 release by multiple cell types including Treg cells with potential to induce peripheral tolerance and suppress ongoing inflammation.

Our study further suggests that the therapeutic effect of oral administration of probiotic is achieved through an IL-10-dependent induction of Tregs. In fact, we were able to prevent EAE in mice by transferring CD4^+^CD25^+^ cells from the probiotic Lacto-mix treated WT mice, but not IL-10 deficient one, showing that probiotic-induced Tregs are tolerogenic once expressing functional IL-10. We also found that animals receiving MLNs from Lacto-mix-treated mice showed increased levels of IL-10 in serum. The connection between the protective activity and Treg cells was further supported by the fact that depletion of CD4^+^ CD25^+^ T cells from the MLN cell population removed the suppressive effect, as well as, excessive serum cytokine levels of IL-10, in EAE recipients.

One issue that remains to be discussed is the strain specific differences on the effect of our probiotic bacteria in EAE. The Lacto-mix strains have previously been isolated from the intestinal mucosa of healthy humans and have been chosen on the basis of their pronounced ability to attach to human mucosa cells [57], [58]. In contrast to *L. paracasei* and *L. plantarum* DSM 15313, the *L. plantarum* DSM 15312 strain binds to epithelial cells via a mannose-dependent mechanism [59]. In addition, a presence of live bacteria in the gut seems to be essential for their immunomodulatory effect since the killed bacterial strains used in our study had no suppressive effect on diseased animals. Our data highlight a major difference even between strains of the same species and the importance of selection of disease specific strains and design a combination of strains with desired properties.

Taken together, we report for the first time that Tregs induced in the gut are able to suppress inflammatory conditions in the periphery, even in the CNS, via an IL-10-dependent mechanism. Thus, induction of Tregs appears to be an important goal of immunotherapy of autoimmune diseases. Our study also provides better understanding of the remarkable immunomodulatory activities of probiotics and a potent synergistic immunosuppressive effect of different probiotic strains for treatment of chronic CNS inflammation which, along with the absence of side-effects, may present a powerful therapeutic approach in the treatment of autoimmune diseases, in general, and MS, in particular.

## Materials and Methods

### Mice

Female C57BL/6 and IL-10-deficient mice on C57BL/6 background (8–10 weeks old) were obtained from Jackson Laboratories (Bar Harbor, ME). The mice were housed in groups in polycarbonate cages with free access to a standard diet and tap water in our animal facility. The trials followed the European Community regulations for animal experiments and were approved by the local Ethical Review Committee for Animal Experiments: Lund's Tingsrätt.

### Induction and Assessment of EAE

A synthetic peptide from myelin oligodendrocyte glycoprotein (MOG), amino acids 35–55 (MEVGWYRSPFSRVVHLYRNGK-COOH, Schafer-N, Denmark) was used to induce EAE. Mice were immunized by an intradermal injection at the base of the tail with 0.1 ml of an emulsion containing 100 µg peptide in complete Freund's adjuvant (H37RA, Difco laboratories, USA), and were injected i.p. with 400 ng of pertussis toxin (Sigma-Aldrich, Sweden) on days 0 and 2. The mice were weighed and examined for clinical signs of EAE in a blinded fashion daily. The signs of EAE were scored into eight categories: 0- no signs of clinical disease; 1- weakness in the tail; 2- paralyzed tail; 3- paresis and gait disturbance; 4- paralysis of one limb; 5- paralysis of two limbs; 6- two limbs paralyzed and paresis of a third limb, but the mouse still able to move forward; 7- quadriplegia, no mobility and moribund state; 8- dead. Food was placed on the cage floor when a mouse showed a score of 5 or higher. To avoid dehydration, mice scored with 6 were subcutaneously given 0.5 ml of physiologic saline solution. When a mouse was scored with 7, it was sacrificed for ethical reasons. At the end of the experiments, the animals were anesthetized, bled by heart puncture and different organs were dissected.

### Bacterial Strains and Treatment

*Lactobacillus paracasei*, DSM 13434 ( = 8700∶2); *Lactobacillus plantarum*, DSM 15312 (HEAL9); *Lactobacillus plantarum*, DSM 15313 (HEAL19); *Lactobacillus paracasei*, PCC (Probi Culture Collection) 101 and *Lactobacillus delbrueckii*, subsp. *bulgaricus*, DSM 20081 (all strains were provided by Probi AB, Lund, Sweden). *Prophylactic treatment* (12 days before the immunization and throughout the experiment): Mice received regular tap water (100 ml in flasks) with the bacteria added to a final concentration of 10^9^ colony-forming units/ml (cfu/ml), while control mice only received tap water. Each mouse drank approximately 5 ml water/day. After the onset of EAE, each animal was fed daily, via an intragastric stainless steel feeding tube, with 200 µl of lactobacilli (10^9^ cfu) or sterile physiological saline (control). The dose of 10^9^ viable cells was chosen as the optimal dose for these investigations and comparable to other probiotic studies in rodents and humans (10^6^–10^12^ cfu/day). *Therapeutic treatment*: Two weeks after disease onset, animals were fed via an intragastric feeding tube with 200 µl of bacterial strains (10^9^ cfu) or saline (control). The diseased animals were only treated every second day to minimize the stress induced by the oral gavage treatment. Instead of live bacteria, groups of animals received a mixture of heat-killed (in sterile normal saline at 100°C for 30 min) bacteria.

### *In Vitro* T Cell Proliferative Response

Spleens and MLNs were harvested and passed through a cell strainer (BD Biosciences, USA). Cell suspensions from spleens were treated with 0.84% NH_4_Cl to lyse the red blood cells. Isolated cells were cultured in round-bottom 96-well culture plates (5×10^5^/well) and stimulated with MOG_35–55_ peptide at 50 µg/ml, using purified protein derivate (PPD) from *Mycobacterium tuberculosis* (Statens Serum Institut, Denmark) as the positive control, at 10 µg/ml (all in triplicates), for 72 h with additional 16 h after addition of 1 µCi of [^3^H]thymidine. Cells were then harvested and counts per minute (CPM) were determined by a gas-flow beta counter (Matrix 96 Direct beta counter; Packard, USA). Stimulation index was calculated by dividing the MOG_35–55_-specific proliferation by proliferation in cultures with medium only.

### ELISA for Cytokine Detection

Cytokine concentrations were measured in serum or supernatants from spleen and MLN cell cultures after *in vitro* challenge with either 50 µg/ml MOG_35–55_ peptide (cells from immunized mice) or 0.5 µg/ml anti-CD3ε (clone 145-2C11, BD Biosciences, USA) monoclonal antibody (cells from non-immunized mice) using commercially available specific ELISA kits. Supernatants were collected after 48 h for measurement of TNF-α, IFN-γ, IL-4, IL-10 (BD OptEIA™ ELISA Sets, BD Biosciences, USA), IL-17A (R&D Systems, UK) and after 72 h for measurement of TGF-β1 and IL-27p28 concentrations using the quantikine kits (R&D Systems, UK). The absorbance was measured in an ELISA reader (Spectra Max M2, Molecular Devices, USA). The cytokine content in supernatants was determined when data were within the linear region of the standard curve calculated from values of the recombinant cytokines.

### Analysis by Flow Cytometry

At the end of each experiment MLNs, spleen and brain were dissected. Single cell suspensions were obtained from MLN and spleen. Brain mononuclear cells were isolated by Percoll gradients as described [33]. Cells were incubated with anti-CD16/CD32 followed by APC/FITC/PE-labelled monoclonal antibodies (mAbs) directed to different murine cell surface markers including CD3, CD4 and CD25 (all purchased from BD Biosciences, USA). Foxp3 was analysed using the PE anti-mouse Foxp3 Staining Set (eBioscience, USA). For analysis of intracellular IL-10 and IL-17A, immune cells were fixed with Cytofix/Cytoperm solution and stained with APC-labelled mAbs directed to different cytokines, (BD Biosciences, USA). Flow-cytometric analysis was performed according to standard settings on a FACSort flow cytometer (Becton Dickinson, USA).

### Immunohistochemistry

Cryosections from the lumbar region of spinal cord were prepared, fixed in acetone, blocked for endogenous avidin/biotin activity (Vector, USA) and incubated with the primary antibody, rat anti-mouse CD4 (BD Biosciences, USA), for 1 h. The secondary antibody, a biotinylated goat anti-rat IgG (Jackson's Immunoresearch, USA) was applied for 30 minutes. After incubation in streptavidin-biotin/peroxidase (Dako, Denmark) for 30 minutes, the immunoreaction was developed in diaminobenzidine (Sigma-Aldrich, Sweden) and the sections were counterstained in haematoxylin.

Brain tissue, including medulla oblongata, was sampled by dividing the organ midsagitally and sectioned parallel to the midline, fixed in 4% formalin, dehydrated, embedded in paraffin, and sectioned at 4 µm. The sections were deparaffinised and incubated with primary antibody rat anti-mouse Foxp3 (eBioscience, CA). Further staining was performed using “EnVision™ FLEX, High pH visualization kit” and “Dako Autostainer Plus” (Dako, Copenhagen, Denmark). The degree of leukocyte infiltration was calculated by using a PC-based image analysis system (Leica Q500, Cambridge, UK). With this equipment, the area of stained “positive” cells was measured and compared to the entire analysed area, as described previously [60].

### Adoptive Transfer of MLN Cells

Naïve female C57BL/6 wild type (WT) or IL-10-deficient (KO) mice were fed with either lactobacilli or sterile saline as described above during 14 days. MLNs were collected and single cell suspensions were prepared and washed extensively. 3×10^5^ cells were then administered intravenously into recipient mice. EAE was induced 24 hours later and the animals were scored for clinical symptoms as mentioned above. In some experiments, CD4^+^CD25^+^ T cells were isolated using a MACS CD4^+^CD25^+^ Regulatory T cell Isolation Kit (Miltenyi Biotech, Germany), performed according to manufacture's instructions.

### Statistical Evaluations

Statistical evaluation was performed using StatView software (SAS, Cary, NC, USA). Student's *t* test was used to analyze the significance of results. Mann-Whitney was used for analyzing differences in clinical scores.
